# Supplementary material for: Autozygosity influences cardiometabolic disease-associated traits in the AWI-Gen sub-Saharan African study
Source: Nat Commun. 2020 Nov 13;11:5754. doi: 10.1038/s41467-020-19595-y (PMC7666169; doi:10.1038/s41467-020-19595-y)

Supplementary Tables and Figures

Supplementary Table 1. β_{FROH} and $\beta_{Foutside\ ROH}$ estimates for the AWI-Gen cohort. Samples sizes (N), inbreeding depression effect size estimates (Beta), standard errors (SE) and associated p-values are shown for the genomic inbreeding coefficient with ROH longer than 1.5Mb (F_{ROH}) and for the homozygosity outside ROH ($F_{outside\ ROH}$). The whole AWI-Gen cohort is represented in this table, results are provided for the whole population (All) and by sex.

Phenotype		N		F_{ROH}			$F_{outside\_ROH}$	
			Beta	SE	P	Beta	SE	P
**SES**	All	10191	-7.19	1.92	1.83E-04	-2.86	7.15	6.89E-01
	Men	4680	-7.15	2.60	6.04E-03	-17.14	10.96	1.18E-01
	Women	5511	-7.23	2.84	1.10E-02	-11.23	9.25	2.25E-01
**Height**	All	10174	-0.13	0.10	1.66E-01	0.45	0.35	2.00E-01
	Men	4676	0.09	0.14	5.18E-01	0.22	0.57	7.00E-01
	Women	5498	-0.39	0.13	3.51E-03	0.58	0.44	1.85E-01
**Weight**	All	10172	-80.09	21.88	2.53E-04	-95.75	81.03	2.37E-01
	Men	4675	-197.47	28.08	2.31E-12	-4.57	108.23	9.66E-01
	Women	5497	60.62	33.71	7.22E-02	-122.25	109.86	2.66E-01
**BMI**	All	10171	-27.75	7.96	4.95E-04	-60.50	29.49	4.02E-02
	Men	4675	-83.68	9.52	2.15E-18	-8.97	34.88	7.97E-01
	Women	5496	39.56	12.79	1.99E-03	-76.92	41.61	6.46E-02
**Waist/Hip**	All	10157	0.28	0.19	1.37E-01	0.17	0.70	8.07E-01
	Men	4670	0.14	0.24	5.70E-01	-0.62	1.12	5.80E-01
	Women	5487	0.46	0.30	1.22E-01	0.49	0.89	5.86E-01
**Visceral Fat**	All	9786	-4.35	2.60	9.35E-02	-2.34	9.55	8.07E-01
	Men	4541	-0.66	3.43	8.48E-01	-0.36	14.06	9.80E-01
	Women	5245	-9.05	3.95	2.20E-02	-5.29	12.62	6.75E-01
**Subcutaneal Fat**	All	9887	-7.29	1.53	2.04E-06	12.21	10.23	2.32E-01
	Men	4597	-16.42	1.90	8.74E-18	28.45	14.58	1.78E-02
	Women	5290	4.35	2.43	7.38E-02	5.47	7.61	4.72E-01
**HDL**	All	10660	-0.86	0.58	1.38E-01	1.52	2.15	4.79E-01
	Men	4852	-2.13	0.85	1.22E-02	0.62	3.58	8.63E-01
	Women	5808	0.52	0.79	5.07E-01	1.92	2.60	4.60E-01
**LDL**	All	10227	-1.64	1.36	2.28E-01	3.88	4.96	4.35E-01
	Men	4615	-8.55	2.04	2.93E-05	7.24	7.83	3.55E-01
	Women	5612	4.70	1.83	1.02E-02	2.22	6.17	7.19E-01
**Cholesterol**	All	10658	-1.39	1.46	3.41E-01	1.55	5.42	7.74E-01
	Men	4852	-6.94	2.05	7.03E-04	0.98	8.39	9.07E-01
	Women	5806	4.80	2.08	2.13E-02	3.55	6.89	6.06E-01
**Triglycerids**	All	10644	-2.16	0.73	3.21E-03	-2.59	2.72	3.42E-01
	Men	4840	-3.30	1.05	1.72E-03	-3.40	4.45	4.44E-01
	Women	5804	-0.95	1.03	3.55E-01	-0.74	3.35	8.26E-01
**Pulse**	All	10280	29.55	17.33	8.83E-02	19.22	63.81	7.63E-01
	Men	4662	17.44	24.78	4.82E-01	-39.43	103.02	7.02E-01
	Women	5618	43.10	24.43	7.77E-02	49.26	80.08	5.39E-01
**Systolic BP**	All	10291	-8.96	29.05	7.58E-01	17.44	107.06	8.71E-01
	Men	4663	4.31	38.87	9.12E-01	19.73	159.32	9.01E-01
	Women	5628	-23.66	43.38	5.86E-01	18.67	143.47	8.96E-01
**Dyastolic BP**	All	10293	8.46	17.83	6.35E-01	-67.32	65.72	3.06E-01
	Men	4661	33.46	24.46	1.71E-01	-39.96	98.99	6.86E-01
	Women	5632	-20.51	26.10	4.32E-01	-90.02	87.57	3.04E-01

Supplementary Table 2. β_{FROH} and $\beta_{Foutside\ ROH}$ estimates for sites with an average night-light intensity below 5. Sample sizes**,** effect size estimates, standard errors and associated p-values are shown for the genomic inbreeding coefficient with ROH longer than 1.5Mb (F_{ROH}) and for the homozygosity outside ROH ($F_{outside\ ROH}$). The AWI-Gen study sites with an average night-light intensity below 5 are represented in this table: Nanoro (Burkina Faso), Navrongo (Ghana), Dikgale and Agincourt (South Africa). Results are provided for the whole population (All) and by sex.

**Phenotype**		**N**		**F_{ROH}**			**$F_{outside\_ROH}$**	
			Beta	SE	P	Beta	SE	P
**SES**	All	6834	-7.30	2.38	2.17E-03	-8.71	12.81	4.96E-01
	Men	2949	-10.29	3.31	1.88E-03			
	Women	3885	-4.05	3.44	2.39E-01			
**Height**	All	6763	-0.10	0.12	3.82E-01	0.08	0.62	8.97E-01
	Men	2943	0.07	0.17	6.66E-01			
	Women	3820	-0.30	0.16	7.10E-02			
**Weight**	All	6762	-99.25	25.08	7.67E-05	-165.22	133.86	2.17E-01
	Men	2943	-280.02	32.25	6.34E-18			
	Women	3819	107.34	38.05	4.82E-03			
**BMI**	All	6761	-37.92	9.04	2.80E-05	-84.68	48.26	7.94E-02
	Men	2943	-119.20	10.92	3.13E-27			
	Women	3818	55.64	14.16	8.63E-05			
**Waist/Hip**	All	6755	0.28	0.20	1.63E-01	-0.31	1.08	7.73E-01
	Men	2939	-0.13	0.26	5.99E-01			
	Women	3816	0.79	0.31	1.26E-02			
**Visceral Fat**	All	6441	-5.84	3.05	5.56E-02	-15.32	16.26	3.46E-01
	Men	2817	-6.69	3.95	9.02E-02			
	Women	3624	-4.80	4.67	3.05E-01			
**Subcutaneal Fat**	All	6537	-13.34	1.89	2.07E-12	9.66	10.01	3.35E-01
	Men	2870	-24.97	2.40	7.71E-25			
	Women	3667	0.63	2.92	8.29E-01			
**HDL**	All	7150	-0.99	0.66	1.32E-01	1.74	3.52	6.21E-01
	Men	3122	-2.21	0.99	2.48E-02			
	Women	4028	0.22	0.89	8.05E-01			
**LDL**	All	6887	-3.76	1.55	1.49E-02	-6.28	8.40	4.55E-01
	Men	2970	-10.45	2.22	2.71E-06			
	Women	3917	3.39	2.16	1.16E-01			
**Cholesterol**	All	7150	-3.09	1.70	6.98E-02	-5.48	9.10	5.47E-01
	Men	3122	-9.27	2.38	1.02E-04			
	Women	4028	3.48	2.44	1.53E-01			
**Triglycerids**	All	7141	-3.89	0.84	4.05E-06	-1.81	4.51	6.88E-01
	Men	3115	-4.32	1.21	3.55E-04			
	Women	4026	-3.52	1.18	2.92E-03			
**Pulse**	All	6735	39.73	20.88	5.71E-02	-5.28	111.54	9.62E-01
	Men	2930	10.87	29.96	7.17E-01			
	Women	3805	71.11	29.40	1.56E-02			
**Systolic BP**	All	6751	-22.08	34.97	5.28E-01	68.29	186.79	7.15E-01
	Men	2931	19.55	47.32	6.80E-01			
	Women	3820	-67.43	51.64	1.92E-01			
**Dyastolic BP**	All	6748	9.92	20.54	6.29E-01	7.17	109.78	9.48E-01
	Men	2929	38.07	28.72	1.85E-01			
	Women	3819	-21.93	29.58	4.58E-01			

Supplementary Table 3. **β_{FROH} and $\beta_{Foutside\ ROH}$ estimates for sites with an average night-light intensity above 5.** Sample sizes**,** effect size estimates, standard errors and associated p-values are shown for the genomic inbreeding coefficient with ROH longer than 1.5Mb (F_{ROH}) and for the homozygosity outside ROH ($F_{outside\ ROH}$). The AWI-Gen study sites with an average night-light intensity above 5 are represented in this table: Nairobi (Kenya) and Soweto (South Africa). Results are provided for the whole population (All) and by sex.

**Phenotype**		**N**	**F_{ROH}**			**$F_{outside\_ROH}$**		
			Beta	SE	P	Beta	SE	P
**SES**	All	3411	-7.48	3.18	1.87E-02	2.46	8.33	7.68E-01
	Men	1733	0.63	4.30	8.83E-01	-3.25	3.12	5.23E-01
	Women	1678	-18.44	4.75	1.07E-04	-4.51	5.12	6.48E-01
**Height**	All	3411	-0.19	0.16	2.27E-01	0.44	0.45	3.20E-01
	Men	1733	0.14	0.22	5.35E-01	0.12	0.21	4.32E-01
	Women	1678	-0.64	0.23	5.72E-03	0.75	0.54	5.89E-01
**Weight**	All	3410	-34.71	40.96	3.97E-01	-30.85	113.32	7.85E-01
	Men	1732	-13.95	50.44	7.82E-01	-5.25	98.12	9.25E-01
	Women	1678	-65.31	66.91	3.29E-01	-84.31	125.23	8.12E-01
**BMI**	All	3410	-6.13	14.92	6.81E-01	-20.87	41.26	6.13E-01
	Men	1732	-10.97	16.85	5.15E-01	-12.12	10.50	5.63E-01
	Women	1678	-1.36	25.84	9.58E-01	-52.12	70.23	7.45E-01
**Waist/Hip**	All	3402	0.44	0.41	2.74E-01	0.37	1.11	7.41E-01
	Men	1731	1.03	0.50	3.87E-02	-0.09	1.23	4.81E-01
	Women	1671	-0.35	0.67	6.04E-01	0.32	2.20	8.02E-01
**Visceral Fat**	All	3345	0.22	4.68	9.63E-01	6.63	12.82	6.05E-01
	Men	1724	10.88	6.36	8.73E-02	0.21	4.23	5.97E-01
	Women	1621	-15.45	6.95	2.64E-02	-1.23	6.23	4.06E-01
**Subcutaneal Fat**	All	3350	5.55	2.43	2.25E-02	-8.44	6.66	2.21E-01
	Men	1727	-0.49	2.88	8.65E-01	4.32	3.12	4.00E-01
	Women	1623	14.25	4.15	6.12E-04	-0.54	1.54	8.17E-01
**HDL**	All	3510	-0.38	1.13	7.35E-01	0.15	3.15	9.62E-01
	Men	1730	-1.41	1.59	3.78E-01	0.10	1.58	7.38E-01
	Women	1780	0.97	1.61	5.46E-01	0.45	6.98	9.10E-01
**LDL**	All	3340	5.33	2.78	5.54E-02	11.49	6.71	8.68E-02
	Men	1645	1.74	4.80	7.18E-01	10.58	8.45	2.97E-01
	Women	1695	7.35	3.35	2.85E-02	2.25	2.78	6.01E-01
**Cholesterol**	All	3508	2.96	2.74	2.79E-01	2.90	7.62	7.04E-01
	Men	1730	-0.43	3.79	9.11E-01	1.02	0.91	4.60E-01
	Women	1778	7.21	3.97	6.92E-02	3.89	2.58	5.01E-01
**Triglycerids**	All	3503	1.54	1.41	2.75E-01	0.23	3.92	9.54E-01
	Men	1725	-1.00	1.98	6.13E-01	-1.23	2.01	6.08E-01
	Women	1778	4.84	2.01	1.60E-02	-2.58	3.78	2.21E-01
**Pulse**	All	3545	11.52	30.34	7.04E-01	-30.19	83.79	7.19E-01
	Men	1732	29.23	42.57	4.92E-01	-45.12	50.89	8.22E-01
	Women	1813	-9.99	43.46	8.18E-01	19.21	10.39	5.18E-01
**Systolic BP**	All	3540	53.30	51.45	3.00E-01	-64.50	142.15	6.50E-01
	Men	1732	15.31	67.63	8.21E-01	-15.25	25.12	8.02E-01
	Women	1808	99.78	78.73	2.05E-01	-3.65	8.68	7.12E-01
**Dyastolic BP**	All	3545	8.43	34.10	8.05E-01	-72.89	94.18	4.39E-01
	Men	1732	30.96	45.22	4.94E-01	-38.08	25.79	6.31E-01
	Women	1813	-20.55	51.78	6.92E-01	-102.25	94.10	5.42E-01

Supplementary Table 4. Effect estimates from bivariate model of Trait ~ F_{ROH} + F_{GRM}. Sample sizes**,** effect size estimates, standard errors and associated p-values are shown for the genomic inbreeding coefficient with ROH longer than 1.5Mb (F_{ROH}) and for the inbreeding coefficient using PLINK's –ibc (F_{GRM}). Results are provided for the whole population (All) and by sex.

**Phenotype**		**N**		**F_{ROH}**			**F_{GRM}**	
			Beta	SE	P	Beta	SE	P
**SES**	All	10191	-5.99	2.50	1.67E-02	-1.30	1.75	4.59E-01
	Men	4882	0.04	3.68	9.92E-01	-3.54	2.74	1.97E-01
	Women	5744	-7.78	3.39	2.16E-02	-2.57	2.26	2.54E-01
**Height**	All	10174	-0.18	0.12	1.45E-01	0.05	0.09	5.37E-01
	Men	4676	-0.06	0.19	7.61E-01	0.05	0.14	7.45E-01
	Women	5498	-0.34	0.16	3.57E-02	0.04	0.11	6.91E-01
**Weight**	All	10172	-92.11	28.61	1.29E-03	13.04	20.00	5.14E-01
	Men	4675	-52.61	16.52	1.21E-03	35.82	27.17	1.87E-01
	Women	5497	-116.14	38.92	1.55E-03	-6.10	26.80	8.20E-01
**BMI**	All	10171	-28.68	10.41	5.89E-03	1.01	7.28	8.89E-01
	Men	4675	-16.80	10.77	1.53E-02	10.95	8.76	2.11E-01
	Women	5496	-29.45	14.50	2.47E-03	-7.43	10.15	4.64E-01
**Waist/Hip**	All	10157	0.32	0.25	2.03E-01	-0.04	0.17	8.36E-01
	Men	4675	-52.61	36.52	1.50E-01	35.82	27.17	1.87E-01
	Women	5487	0.37	0.33	2.72E-01	0.11	0.22	6.21E-01
**Visceral Fat**	All	9786	-4.93	3.39	1.46E-01	0.62	2.34	7.92E-01
	Men	4541	-2.91	4.78	5.42E-01	2.90	3.54	4.12E-01
	Women	5245	-5.65	4.74	2.34E-01	-1.35	3.04	6.58E-01
**Subcutaneal**	All	9887	-15.80	1.99	2.42E-15	-9.18	4.38	2.67E-04
	Men	4597	-18.72	2.56	3.24E-13	-11.58	5.90	1.79E-05
	Women	9887	15.80	1.99	2.42E-15	9.18	3.38	2.67E-04
**HDL**	All	10660	-0.65	0.75	3.87E-01	-0.23	0.53	6.70E-01
	Men	4852	-0.93	1.20	4.36E-01	-0.52	0.90	5.64E-01
	Women	5808	-2.01	1.25	3.21E-02	0.56	1.52	7.14E-01
**LDL**	All	10227	-2.19	1.75	2.12E-01	0.62	1.25	6.21E-01
	Men	4615	-0.76	0.67	7.75E-02	-0.51	2.02	8.00E-01
	Women	5612	2.01	0.95	3.72E-03	0.56	1.52	7.14E-01
**Cholesterol**	All	10658	-1.17	1.90	5.38E-01	-0.24	1.34	8.59E-01
	Men	4841	-1.42	1.49	3.40E-01	0.27	1.11	8.06E-01
	Women	5806	-2.00	2.53	4.31E-01	0.43	1.69	8.01E-01
**Triglycerids**	All	10644	-2.69	0.66	1.83E-03	-0.59	0.67	3.84E-01
	Men	4841	-2.42	0.49	3.40E-04	-0.27	1.11	8.06E-01
	Women	5805	-3.44	1.23	5.31E-03	0.84	0.82	3.07E-01
**Pulse**	All	10280	25.80	22.69	2.56E-01	4.06	15.88	7.98E-01
	Men	4662	11.56	34.79	7.40E-01	-8.22	25.84	7.50E-01
	Women	5618	48.34	30.13	1.09E-01	12.11	19.82	5.41E-01
**Systolic BP**	All	10291	-23.74	38.03	5.32E-01	16.04	26.63	5.47E-01
	Men	4663	-4.47	53.75	9.34E-01	24.80	40.00	5.35E-01
	Women	5628	-57.33	53.94	2.88E-01	4.75	35.48	8.94E-01
**Dyastolic BP**	All	10293	12.07	23.35	6.05E-01	-3.92	16.35	8.11E-01
	Men	4661	27.02	33.39	4.18E-01	5.74	24.85	8.17E-01
	Women	5632	-7.77	32.94	8.14E-01	-13.29	21.67	5.39E-01

Supplementary Table 5. **Protein coding genes present in the genomic window exhibiting an inbreeding depression effect on BMI in men**. Chromosome, start and end, gene name, Ensembl gene ID and a short description is given for every protein coding gene present in the window.

Chr.	Start	End	Gene	Ensembl gene Id	Description
14	62699454	63102037	KCNH5	ENSG00000140015	Potassium voltage-gated channel subfamily
14	63204114	63293219	RHOJ	ENSG00000126785	Ras homolog family member J
14	63312835	63318879	GPHB5	ENSG00000179600	Glycoprotein hormone beta 5
14	63371357	63543374	PPP2R5E	ENSG00000154001	Protein phosphatase 2 regulatory subunit B'epsilon
14	63597039	63641861	WDR89	ENSG00000140006	WD repeat domain 89
14	63684214	63728039	SGPP1	ENSG00000126821	Sphingosine-1-phosphate phosphatase 1
14	63852983	64226433	SYNE2	ENSG00000054654	Spectrin repeat containing nuclear envelope protein 2
14	64084232	64338112	ESR2	ENSG00000140009	Estrogen receptor 2
14	64388031	64463457	MTHFD1	ENSG00000100714	Methylenetetrahydrofolate dehydrogenase,
14	64449106	64505213	ZBTB25	ENSG00000089775	Zinc finger and BTB domain containing 25
14	64465499	64474503	AKAP5	ENSG00000179841	A-kinase anchoring protein 5
14	64503712	64533690	ZBTB1	ENSG00000126804	Zinc finger and BTB domain containing 1
14	64535905	64546173	HSPA2	ENSG00000126803	Heat shock protein family A (Hsp70) member 2
14	64549902	64589380	PPP1R36	ENSG00000165807	Protein phosphatase 1 regulatory subunit 36
14	64704102	64750247	PLEKHG3	ENSG00000126822	Pleckstrin homology and RhoGEF domain containing G3
14	64746283	64879907	SPTB	ENSG00000070182	Spectrin beta, erythrocytic
14	64914361	64944591	CHURC1	ENSG00000258289	Churchill domain containing 1
14	64914485	65061803	CHURC1-	ENSG00000125954	CHURC1-FNTB readthrough
14	64939152	64942905	GPX2	ENSG00000176153	Glutathione peroxidase 2
14	64945814	64973226	RAB15	ENSG00000139998	RAB15, member RAS oncogene family
14	64986720	65062652	FNTB	ENSG00000257365	Farnesyltransferase, CAAX box, beta
14	65006174	65102695	MAX	ENSG00000125952	MYC associated factor X
14	65410592	65744121	FUT8	ENSG00000033170	Fucosyltransferase 8

Supplementary Table 6. **Protein coding genes present in the genomic windows exhibiting an inbreeding depression effect on VAT in women**. Chromosome, start and end, gene name, Ensembl gene ID and a short description is given for every protein coding gene present in the two windows.

Chr.	Start	End	Gene	Ensembl gene Id	Description
**2**	38814	46870	FAM110C	ENSG00000184731	Family with sequence similarity 110 member C
**2**	217730	266398	SH3YL1	ENSG00000035115	SH3 and SYLF domain containing 1
**2**	264140	278283	ACP1	ENSG00000143727	Acid phosphatase 1
**2**	279558	288851	ALKAL2	ENSG00000189292	ALK and LTK ligand 2
**2**	667335	677439	TMEM18	ENSG00000151353	Transmembrane protein 18
**2**	950868	1367613	SNTG2	ENSG00000172554	Syntrophin gamma 2
**2**	1374066	1543711	TPO	ENSG00000115705	Thyroid peroxidase
**2**	1631887	1744852	PXDN	ENSG00000130508	Peroxidasin
**2**	1789113	2331664	MYT1L	ENSG00000186487	Myelin transcription factor 1 like
**4**	56977722	57031168	POLR2B	ENSG00000047315	RNA polymerase II subunit B
**4**	57030773	57110385	IGFBP7	ENSG00000163453	Insulin like growth factor binding protein 7

Supplementary Table 7. **Median and interquartile range (IQR) of the genomic inbreeding coefficient (F_{ROH}) in sampling sites.**

Site	F_{ROH}	
	**Median**	**IQR**
**Agincourt**	0.0072	0.0042
**Dikgale**	0.0062	0.0041
**Nairobi**	0.0043	0.0039
**Soweto**	0.0061	0.0037
**Nanoro**	0.0026	0.0031

Supplementary Figure 1. **Q-Q plots. a**: Inbreeding depression on body mass index (BMI) for men. **b**: Inbreeding depression on visceral adipose tissue (VAT) for women.

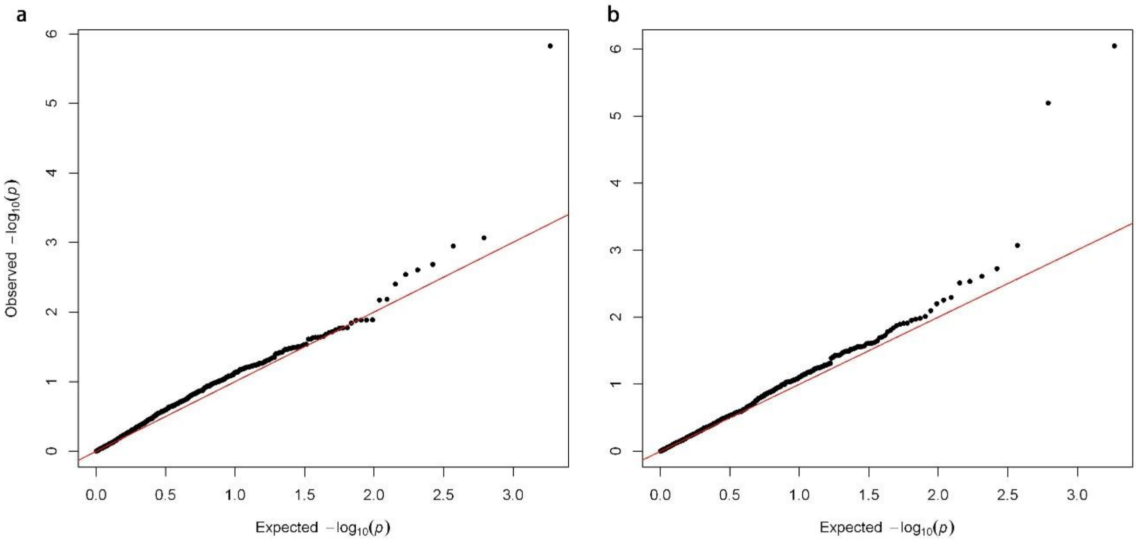

Supplement: Supplementary file 1 — Supplementary Information [file 41467_2020_19595_MOESM1_ESM.pdf]
