# Supplementary material for: Autozygosity influences cardiometabolic disease-associated traits in the AWI-Gen sub-Saharan African study
Source: Nat Commun. 2020 Nov 13;11:5754. doi: 10.1038/s41467-020-19595-y (PMC7666169; doi:10.1038/s41467-020-19595-y)
Supplement: Supplementary file 3 — Reporting Summary [file 41467_2020_19595_MOESM3_ESM.pdf]

nature research

Corresponding author(s): ceballoscamina@gmail.com

Last updated by author(s): May 19, 2020

Reporting Summary

Nature Research wishes to improve the reproducibility of the work that we publish. This form provides structure for consistency and transparency in reporting. For further information on Nature Research policies, see Authors & Referees and the Editorial Policy Checklist.

Statistics

For all statistical analyses, confirm that the following items are present in the figure legend, table legend, main text, or Methods section.

n/a	Confirmed	
☐	☒	The exact sample size (*n*) for each experimental group/condition, given as a discrete number and unit of measurement
☐	☒	A statement on whether measurements were taken from distinct samples or whether the same sample was measured repeatedly
☐	☒	The statistical test(s) used AND whether they are one- or two-sided<br>*Only common tests should be described solely by name; describe more complex techniques in the Methods section.*
☐	☒	A description of all covariates tested
☐	☒	A description of any assumptions or corrections, such as tests of normality and adjustment for multiple comparisons
☐	☒	A full description of the statistical parameters including central tendency (e.g. means) or other basic estimates (e.g. regression coefficient) AND variation (e.g. standard deviation) or associated estimates of uncertainty (e.g. confidence intervals)
☐	☒	For null hypothesis testing, the test statistic (e.g. *F*, *t*, *r*) with confidence intervals, effect sizes, degrees of freedom and *P* value noted<br>*Give P values as exact values whenever suitable.*
☒	☐	For Bayesian analysis, information on the choice of priors and Markov chain Monte Carlo settings
☒	☐	For hierarchical and complex designs, identification of the appropriate level for tests and full reporting of outcomes
☐	☒	Estimates of effect sizes (e.g. Cohen's *d*, Pearson's *r*), indicating how they were calculated

Our web collection on statistics for biologists contains articles on many of the points above.

Software and code

Policy information about availability of computer code

Data collection	Africa Wits-INDEPTH Partnership for Genomic Studies (AWI-Gen) is an NIH-funded Collaborative Centre of the Human Heredity and Health in Africa (H3Africa) Consortium. Data gathered by the AWI-Gen initiative was QC'ed and analysed by a suite of custom R scripts (version 3.2.6 or later).
Data analysis	Analyses of the AWI-Gen cohort were performed using R scripts (version 3.2.6 or later) developed by the ROHgen team, the analysis scripts made external calls to commercial software programs PLINK (v1.9 or later) and KING (v2 or later) for specific requirements.

For manuscripts utilizing custom algorithms or software that are central to the research but not yet described in published literature, software must be made available to editors/reviewers. We strongly encourage code deposition in a community repository (e.g. GitHub). See the Nature Research guidelines for submitting code & software for further information.

Data

Policy information about availability of data

All manuscripts must include a data availability statement. This statement should provide the following information, where applicable:

- Accession codes, unique identifiers, or web links for publicly available datasets
- A list of figures that have associated raw data
- A description of any restrictions on data availability

The data phenotype and genetic will be submitted to the European Genome-phenome Archive (EBI) under the H3Africa Consortium and AWI-Gen. Data are available on application to the H3Africa Data and Biospecimen Access Committee or through collaboration with the authors.

Field-specific reporting

Please select the one below that is the best fit for your research. If you are not sure, read the appropriate sections before making your selection.

☒ Life sciences ☐ Behavioural & social sciences ☐ Ecological, evolutionary & environmental sciences

For a reference copy of the document with all sections, see nature.com/documents/nr-reporting-summary-flat.pdf

Life sciences study design

All studies must disclose on these points even when the disclosure is negative.

Sample size	The AWI-Gen cohort has in total over 12,500 participants recruited between August 2013 and August 2016, of which 10,776 were aged 40 – 60 years. In this analysis after several quality control steps we use a final dataset containing 1,733,121, SNVs and 10,617 individuals. This is the largest sample from Sub-Saharan Africa used up to date. The large sample size used and the large range of the FROH coefficient provide us with enough statistical power to detect those traits with directional dominance architecture.
Data exclusions	Samples where excluded from an analysis when the trait value exceeded pre-defined limits of normal variation. This exclusion removed clearly erroneous values caused by data entry typos. For example, samples with recorded heights <1.0 m or >2.4 m were not included in the analysis of height.
Replication	This study is the first of its kind developed in populations from Sub-Saharan Africa. Even more, this study can be considered a replication in Africa of previous papers that tested the susceptibility of several physiological traits to inbreeding: Joshi et al (2015), Clark et al (2019). Our results are in concordance with previous ones. The statistical methods of linear regression and variance analysis are well established to be unbiased, and the consistent effect estimates across numerous cohorts of different demographic backgrounds reduces the likelihood of confounding.
Randomization	The study design does not allocate samples to groups, but rather uses predefined algorithms to calculate an inbreeding coefficient (FROH) for each individual on a continuous scale from 0 to 1. Randomization is therefore not relevant to the study design, although care was taken to exclude the possibility of spurious associations between the traits of interest and possible confounders. Specifically, covariates were added in the linear regression models including: age, sex, genotyping batch, assessment center, principal components of autosomal genotypes, measures of socio-economic deprivation, etc. Full lists of fitted covariates for each trait are provided in the online methods.
Blinding	The study design does not allocate samples to groups, but rather uses predefined algorithms to calculate an inbreeding coefficient (FROH) for each individual on a continuous scale from 0 to 1. Since the algorithm used to calculate FROH was predefined, investigators were of course completely blind to 'group' allocation. Urbanization and the degree of development of each sampling site were determined by the night-light intensity or luminosity. Luminosity has been extensively used and tested as a proxy for this matter.

Reporting for specific materials, systems and methods

We require information from authors about some types of materials, experimental systems and methods used in many studies. Here, indicate whether each material, system or method listed is relevant to your study. If you are not sure if a list item applies to your research, read the appropriate section before selecting a response.

Materials & experimental systems

n/a	Involved in the study	
☒	☐	Antibodies
☒	☐	Eukaryotic cell lines
☒	☐	Palaeontology
☒	☐	Animals and other organisms
☐	☒	Human research participants
☒	☐	Clinical data

Methods

n/a	Involved in the study	
☒	☐	ChIP-seq
☒	☐	Flow cytometry
☒	☐	MRI-based neuroimaging

Human research participants

Policy information about studies involving human research participants

Population characteristics	Men and women were included in this study. The majority were between 40 and 60 years of age at collection and were recruited from 6 study sites in four African countries as described in the methods. It was a population-cross sectional study and did not select participants based on specific traits.
Recruitment	The study was preceded by community engagement activities (described elsewhere) and following agreement by community leaders for entry into the community for this study, smaller groups were recruited and informed individual broad consent was obtained from each participant. Recruitment was voluntary and no one was disadvantaged if they decided not to take part in the study. Participants were told that they may withdraw their data and samples form the study at any time, but were advised that

once the data had been analysed or submitted to repositories, it would not longer be possible to withdraw that data. All data and samples were anonymised with study codes.

Ethics oversight	This study was approved by the Human Research Ethics Committee (Medical) of the University of the Witwatersrand (Wits) (protocol numbers M121029 and M170880), and each contributing Centre obtained additional local ethics approval, as required.

Note that full information on the approval of the study protocol must also be provided in the manuscript.
